# Supplementary material for: Reversible gating of singlet fission by tuning the role of a charge-transfer state
Source: Nat Commun. 2025 Mar 26;16:2968. doi: 10.1038/s41467-025-58168-9 (PMC11947088; doi:10.1038/s41467-025-58168-9)
Supplement: Supplementary file 1 — Supplementary Information [file 41467_2025_58168_MOESM1_ESM.pdf]

Supporting Information

Reversible gating singlet fission by tuning the role of a charge transfer state

Yifan Bo,[a] Yuxuan Hou,[b,†] Dominic A. X. Lavergne,[b] Timothy Clark,[c] Michael J. Ferguson,[b] Rik R. Tykwinski,[*,b] Dirk M. Guldi[*,a]

[a]Department of Chemistry and Pharmacy & Interdisciplinary Center for Molecular Materials (ICMM), FAU Profile Center Solar, Friedrich-Alexander-Universität Erlangen-Nürnberg, Egerlandstr. 3, 91058 Erlangen, Germany.

[b]Department of Chemistry, University of Alberta, 11227 Saskatchewan Drive, Edmonton, Alberta, Canada T6G 2G2.

[c]Department of Chemistry and Pharmacy & Computer-Chemie-Center (CCC), Friedrich-Alexander-Universität Erlangen-Nürnberg, Nägelsbachstr. 25, 91052 Erlangen, Germany.

[†]Current address: Department of Chemistry, University of Copenhagen, 2100 Copenhagen Ø, Denmark

[*]Corresponding authors

Table of Contents

Supplementary Note 1. Synthesis and Experimental Data S2

Supplementary Note 2. Photophysical Characterization S10

Supplementary Note 3. Supplementary References S23

Supplementary Note 1. Synthesis and Experimental Data

Synthesis of 3,5-bis(4-*tert*-butylphenyl)-4-ethynylpyridine (compound **1**):*

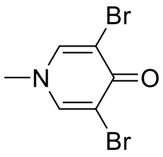

Compound **S1**: The methylation of 4-pyridone was carried out be adapting the procedure of Eidamshaus and Reissig.[1] To the solution of 3,5-dibromo-4-pyridone (3.0 g, 12 mmol) and K_2CO_3 (3.28 g, 23.7 mmol) in dimethylformamide (100 mL) was added methyl iodide (2.52 g, 17.8 mmol). The mixture was stirred at rt for 72 h. NaOH solution (1M, 50 mL) and CH_2Cl_2 (100 mL) were added, the layers were separated, and the aqueous phase was extracted with CH_2Cl_2 (2 × 50 mL). The organic phases were combined, dried ($MgSO_4$), and filtered. Solvent removal and recrystallization from MeOH (20 mL) afforded compound **S1** (2.5 g, 79%) as a white solid. ^{1}H NMR (500 MHz, $CDCl_3$) δ 7.70 (s, 2H), 3.73 (s, 3H). ^{13}C NMR (125 MHz, $CDCl_3$) δ 167.9, 140.4, 113.3, 44.0.

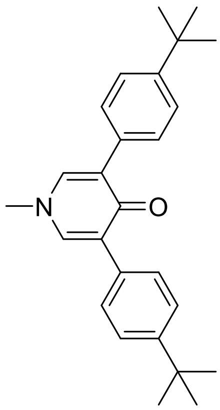

Compound **S2**: A solution of toluene (100 mL) and EtOH (25 mL) was deoxygenated under a flow of N_2 for 20 min. To the solution was added 2-(4-*tert*-butylphenyl)-4,4,5,5-tetramethyl-1,3,2-dioxaborolane (3.50 g, 13.5 mmol), compound S1 (1.20 g, 4.50 mmol), $Pd(PPh_3)_4$ (312 mg, 0.270 mmol), and Cs_2CO_3 (5.86 g, 18.0 mmol). The mixture was stirred for 62 h at 80 °C under a N_2 atmosphere. The reaction mixture was cooled to rt, H_2O (50 mL) and ethyl acetate (40 mL) were added, the layers were separated, and the aqueous phase was extracted with ethyl acetate (2 × 40 mL). The organic phases were combined, washed with satd aq NH_4Cl (2 × 20 mL), dried ($MgSO_4$), and filtered. Solvent removal and purification by column chromatography (silica gel, EtOAc/CH_2Cl_2 1:8) afforded **S2** (1.45 g, 86%) as a white solid. Mp: no visible change ≤ 300 °C. R_f = 0.50 (EtOAc/hexanes 1:1). IR (cast film) 3049 (w), 2966 (s), 2867 (w), 1644 (s), 1559 (s) cm^{-1}. ^{1}H NMR (500 MHz, $CDCl_3$) δ 7.60 (d, J = 8.4 Hz, 4H), 7.41 (d, J = 8.4 Hz, 4H), 7.40 (s, 2H), 3.72 (s, 3H), 1.33 (s, 18H). ^{13}C NMR (125 MHz, $CDCl_3$) δ 174.3, 150.4, 137.6, 132.2, 130.3, 128.4, 125.1, 44.0, 34.6, 31.4. Further spectral data are available in reference 2.[2]

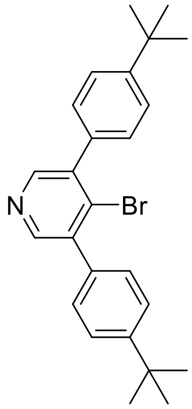

Compound **S3**: To PBr_3 (4 mL) was added S2 (200 mg, 0.535 mmol). The mixture was stirred for 4 h at 150 °C under a N_2 atmosphere. After cooling to rt, the reaction mixture was added dropwise to a NaOH solution (1M, 300 mL) in an ice bath. The mixture was stirred for 5 min. CH_2Cl_2 (50 mL) was added, the layers were separated, and the aqueous phase was extracted with CH_2Cl_2 (3 × 50 mL). The organic phases were combined, washed with satd aq NH_4Cl (2 × 20 mL), dried ($MgSO_4$), and filtered. Solvent removal and purification by column chromatography (silica gel, EtOAc/hexanes 1:10) afforded **S3** (192 mg, 85%) as a white solid. Mp 180–181 °C.

* The synthesis of compound **1** is presented here as adapted from reference [2].

R_f = 0.57 (EtOAc/hexanes 1:4). IR (cast film) 3046 (w), 3025 (w), 2960 (s), 2903 (m), 2868 (m), 1542 (m), 1508 (m) cm^{-1}. ^{1}H NMR (500 MHz, $CDCl_3$) δ 8.43 (s, 2H), 7.50, 7.41 (ABq, J_{AB} = 8.5 Hz, 8H), 1.39 (s, 18H). ^{13}C NMR (125 MHz, $CDCl_3$) δ 151.3, 149.4, 139.0, 135.0, 133.4, 129.3, 125.2, 34.7, 31.3. Further spectral data are available in reference 2.[2]

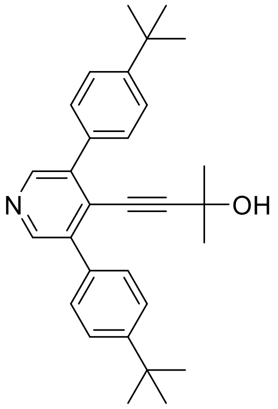

Compound **S4**: 2-Methylbut-3-yn-2-ol (498 mg, 5.92 mmol) was added to a solution of triethylamine (5 mL) and deoxygenated under a flow of N_2 for 20 min. To this solution was added S3 (250 mg, 0.592 mmol), $Pd(PPh_3)_4$ (34 mg, 0.030 mmol), and CuI (17 mg, 0.089 mmol). The mixture was stirred for 2 d at 80 °C under a N_2 atmosphere. The reaction mixture was cooled to rt, H_2O (20 mL) and ethyl acetate (20 mL) were added, the layers were separated, and the aqueous phase was extracted with ethyl acetate (2 × 20 mL). The organic phases were combined, washed with satd aq NH_4Cl (2 × 20 mL), dried ($MgSO_4$), and filtered. Solvent removal and purification by column chromatography (silica gel, EtOAc/hexanes 1:6) afforded **S4** (182 mg, 72%) as a white solid. Mp 124–126 °C. R_f = 0.56 (EtOAc/hexanes 1:2). IR (cast film) 3223 (br, m), 3032 (m), 2964 (s), 2905 (m), 2868 (m), 2228 (w), 1577 (m), 1508 (m) cm^{-1}. ^{1}H NMR (500 MHz, $CDCl_3$) δ 8.58 (s, 2H), 7.54, 7.48 (ABq, J_{AB} = 8.5 Hz, 8H), 1.37 (s, 18H), 1.30 (s, 6H). ^{13}C NMR (125 MHz, $CDCl_3$) δ 151.3, 148.2, 138.4, 134.3, 129.1, 127.4, 125.0, 104.2, 79.2, 65.4, 34.7, 31.3, 30.3. Further spectral data are available in reference 2.[2]

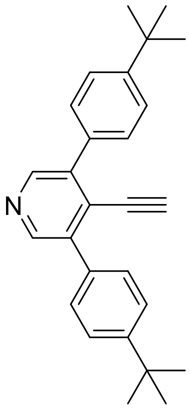

Compound **1**: To a solution of S4 (100 mg, 0.235 mmol) in dried toluene (10 mL) was added well-powdered NaOH (94.0 mg, 2.35 mmol) and TDA-1 (76.0 mg, 0.235 mmol). The solution was stirred for 4 h at rt, only a trace amount of product can be observed as monitored by TLC analysis. The solution was then stirred for 4 h at 110 °C until conversion was judged completely by TLC analysis. The reaction mixture was cooled to rt, H_2O (20 mL) and ethyl acetate (20 mL) were added, the layers were separated, and the aqueous phase was extracted with ethyl acetate (2 × 20 mL). The organic phases were combined, washed with satd aq NH_4Cl (2 × 20 mL), dried ($MgSO_4$), and filtered. Solvent removal and purification by column chromatography (silica gel, EtOAc/hexanes 1:12) afforded **1** (82 mg, 95%) as a white solid. Mp 198–199 °C. R_f = 0.52 (EtOAc/hexanes 1:4). IR (cast film) 3290 (s), 3049 (w), 3029 (w), 2960 (s), 2904 (m), 2866 (m), 2110 (w), 1572 (m), 1506 (m) cm^{-1}. ^{1}H NMR (500 MHz, $CDCl_3$) δ 8.58 (s, 2H), 7.57, 7.49 (ABq, J_{AB} = 8.4 Hz, 8H), 3.21 (s, 1H), 1.38 (s, 18H). ^{13}C NMR (125 MHz, $CDCl_3$) δ 151.2, 148.6, 139.1, 134.0, 129.2, 126.3, 125.2, 88.1, 79.7, 34.7, 31.4. Further spectral data are available in reference 2.[2]

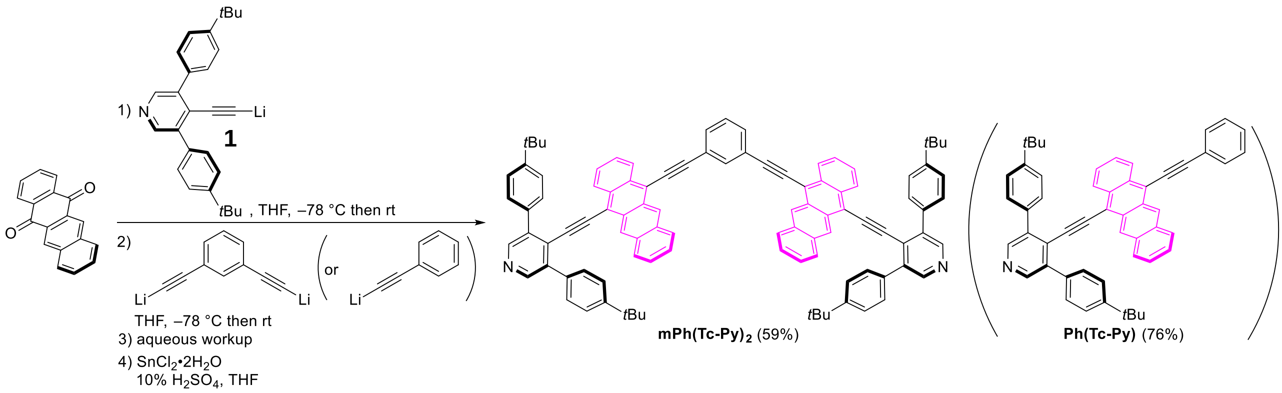

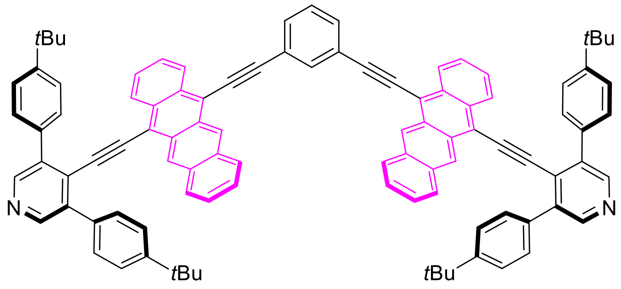

Compound mPh(Tc-Py)$_2$. A solution of lithium hexamethyldisilazide (LiHMDS) was prepared by adding *n*BuLi (2.5 M in hexanes, 1.3 mL, 3.2 mmol) to a solution of $[(CH_3)_3Si]_2NH$ (0.70 mL, 3.3 mmol) in dry THF (5 mL) at –78 °C. The solution was stirred at –78 °C for 30 min, and a solution of **1** (134 mg, 0.36 mmol) in dry THF (10 mL) was added at –78 °C. After stirring at –78 °C for 30 min, the resulting solution was added to a solution of 5,12-naphthacenequinone (86 mg, 0.33 mmol) in dry THF (10 mL) at –78 °C via a cannula. The resulting solution was warmed to rt and stirred for 4 h. After cooling to –78 °C, 1,3-diethynylbenzene† (20 mg, 0.16 mmol) in dry THF (10 mL) was added. The solution was warmed to rt and stirred for 16 h under an atmosphere of N_2. After cooling to 0 °C, satd aq NH_4Cl (10 mL) was added, followed by H_2O (50 mL), the layers were separated, and the aqueous phase was extracted with CH_2Cl_2 (3 × 50 mL). The organic phases were combined, dried ($MgSO_4$), and filtered. After removing the solvent under reduced pressure, the residue was dissolved in CH_2Cl_2 and then passed through a pad of silica gel with EtOAc/hexanes 1:2, followed by solvent removal in vacuo. The crude product mixture was dissolved in dry, deoxygenated THF (20 mL), to which $SnCl_2•2H_2O$ (220 mg, 0.95 mmol) and 10% H_2SO_4 (0.1 mL) were added at rt. The flask was wrapped in aluminum foil during the reaction to limit light exposure. The reaction mixture was stirred for 16 h under an atmosphere of N_2 and then passed through a pad of basic alumina (THF), followed by solvent removal in vacuo. The residue was purified by column chromatography (silica gel, EtOAc/hexanes 1:3) followed by solvent removal in vacuo. And the solid was dissolved in minimal amount of CH_2Cl_2 (~1 mL) followed

† 1,3-Diethynylbenzene is commercially available (CAS Number: 1785-61-1).

by the addition of MeOH (20 mL). The resulting suspension was filtered, and the residue was washed with MeOH (3 × 5 mL), affording mPh(Tc-Py)$_2$ as a dark purple solid (122 mg, 59%). Mp >350 °C (no obvious change). R_f = 0.30 (EtOAc/hexanes 1:3). UV-vis (CH_2Cl_2) λ_{max} (ε) 294 (203000), 337 (28200), 374 (31800), 411 (8470), 465 (sh, 6080), 493 (16800), 528 (41600), 570 nm (64300 $M^{-1}cm^{-1}$). IR (CH_2Cl_2, cast) 3053 (w), 3029 (w), 2962 (s), 2904 (m), 2868 (w), 2180 (w), 1591 (m) cm^{-1}. ^{1}H NMR (498 MHz, $CDCl_3$) δ 9.23 (s, 2H), 8.73 (s, 4H), 8.64 (d, J = 8.9 Hz, 2H), 8.43 (s, 2H), 8.23 (s, 1H), 8.08 (d, J = 8.5 Hz, 2H), 7.87 (dd, J = 8.8 Hz, 1.6 Hz, 2H), 7.82 (d, J = 6.8 Hz, 8H), 7.73 (d, J = 8.7 Hz, 2H), 7.60 (t, J = 7.8 Hz, 1H), 7.55 (d + m, J = 8.5 Hz, 10H), 7.53–7.49 (m, 2H), 7.45–7.38 (m, 4H), 7.23–7.20 (m, 2H), 1.30 (s, 36H). ^{13}C NMR (126 MHz, $CDCl_3$) δ 151.7, 149.0, 138.5, 134.9, 134.6, 132.7, 132.3, 132.20, 132.16, 132.0, 129.9, 129.8, 129.5, 129.3, 129.1, 128.4, 128.0, 127.4, 127.1, 126.71, 126.68, 126.2, 125.9, 125.7, 124.1, 118.7, 118.0, 102.6, 100.4, 97.4, 88.0, 34.8, 31.4. (two signals coincident or not observed). MALDI HRMS calcd for $C_{100}H_{80}N_2$ (M^+) 1308.6316, found 1308.6322. DSC: Decomposition, 264 °C (onset), 325 °C (peak).

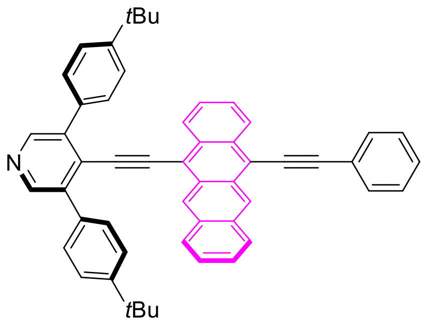

Compound Ph(Tc-Py). A solution of LiHMDS was prepared by adding *n*BuLi (2.5 M in hexanes, 0.88 mL, 2.2 mmol) to a solution of [$(CH_3)_3Si]_2NH$ (0.48 mL, 2.3 mmol) in dry THF (5 mL) at –78 °C. The solution was stirred at –78 °C for 30 min, and a solution of **1** (49 mg, 0.13 mmol) in dry THF (10 mL) was added at –78 °C. After stirring at –78 °C for 30 min, the solution was added to a solution of 5,12-naphthacenequinone (29 mg, 0.11 mmol) in dry THF (10 mL) at –78 °C via a cannula. The solution was warmed to rt and stirred for 4 h. After cooling to –78 °C, 4-ethynylbenzene (17 mg, 0.17 mmol) in dry THF (10 mL) was added. The solution was warmed to rt and stirred for 16 h under an atmosphere of N_2. After cooling to 0 °C, satd aq NH_4Cl (10 mL) was added, followed by H_2O (50 mL), the layers were separated, and the aqueous phase was extracted with CH_2Cl_2 (3 × 50 mL). The organic phases were combined, dried ($MgSO_4$), and filtered. After removing the solvent, the residue was dissolved in CH_2Cl_2 and passed through a pad of silica gel (EtOAc/hexanes 1:2), followed by solvent removal in vacuo. The crude product mixture was dissolved in dry, deoxygenated THF (20 mL), to which $SnCl_2$•$2H_2O$ (74 mg, 0.33 mmol) and 10% H_2SO_4 (0.1 mL) were added at rt. The flask was wrapped in aluminum foil during the reaction to limit light exposure. The reaction mixture was stirred for 16 h under an atmosphere of N_2 and then passed through a pad of basic alumina (THF), followed by solvent removal in vacuo. The residue was purified by column chromatography (silica gel, EtOAc/hexanes 1:3)

followed by solvent removal in vacuo. And the solid was dissolved in minimal amount of CH_2Cl_2 (~1 mL) followed by the addition of MeOH (20 mL). The resulting suspension was filtered, and the residue was washed with MeOH (3 × 5 mL), affording **Ph(Tc-Py)** as a scarlet solid (60 mg, 76%). Decomposition 272 °C. R_f = 0.32 (EtOAc/hexanes 1:3). UV-vis (CH_2Cl_2) λ_{max} (ε) 295 (110000), 322 (13800), 374 (14200), 412 (3500), 463 (sh, 2900), 492 (4830), 525 (21700), 565 nm (31900 $M^{-1}cm^{-1}$). IR (CH_2Cl_2, cast) 3055 (w), 3034 (w), 2965 (s), 2903 (m), 2867 (w), 2189 (m), 1613 (w), 1597 (m), 1569 (s) cm^{-1}. ^{1}H NMR (498 MHz, $CDCl_3$) δ 9.19 (s, 1H), 8.72 (s, 2H), 8.59 (d, J = 8.7 Hz, 1H), 8.41 (s, 1H), 8.03 (d, J = 8.4 Hz, 1H), 7.81 (d, J = 8.4 Hz, 6H), 7.71 (d, J = 8.5 Hz, 1H), 7.56–7.38 (m, 11H), 7.21–7.18 (m, 1H), 1.30 (s, 18H). ^{13}C NMR (126 MHz, $CDCl_3$) δ 151.6, 149.0, 138.5, 134.9, 132.8, 132.11, 132.06, 131.8, 129.9, 129.8, 129.5, 129.2, 128.9, 128.7, 128.4, 128.0, 127.3, 127.2, 126.6, 126.5, 126.1, 125.81, 125.75, 125.6, 123.5, 119.3, 117.5, 103.7, 100.2, 97.5, 87.1, 34.8, 31.4. (two signals coincident or not observed). MALDI HRMS calcd for $C_{53}H_{43}N$ (M^+) 693.3396, found 693.3390. DSC: Decomposition, 273 °C (onset), 280 °C (peak).

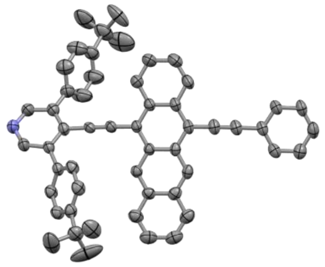

A crystal of Ph(Tc-Py) suitable for crystallographic analysis has been grown at rt by slow evaporation of a $CDCl_3/CH_2Cl_2$ solution layered with MeOH. X-ray data for Ph(Tc-Py) ($C_{53}H_{43}N$), F_w = 693.88; crystal dimensions 0.25 × 0.15 × 0.02 mm, triclinic crystal system; space group $P\bar{1}$ (No. 2); a = 9.5667(3) Å, b = 13.6165(3) Å, c = 15.6536(4) Å; α = 83.3598(16)°, β = 86.0054(16)°, γ = 75.247(2)°; V = 1956.94(9) Å^3; Z = 2; ρ_{calcd} = 1.178 g/cm^3; $2\theta_{max}$ = 140.94°; μ = 0.508 mm^{-1}; T = 173 K; total data collected = 49759; R_1 = 0.0548 [4970 observed reflections with $F_o{}^2 \geq 2\sigma(F_o{}^2)$]; ωR_2 = 0.1705 for 7204 data, 833 variables, and 899 restraints; largest difference, peak and hole = 0.286 and –0.223 e Å^{-3}. A total of 185 same distance (SADI) restraints were applied to the following sets of atoms to improve the geometry of the minor component of the nearly whole-molecule disorder: C3–C26 and C3–C26A; the C–C distances within the *tert*-butyl group containing atoms C12 to C15A; the C–C distances within the phenyl group C16 to C20 and C16A to C20A; C19–C22 and C19A–C22A; the C–C distances within the *tert*-butyl group containing atoms C22 to C25 and C22A to C25A; the C19–C22 and C19A–C22A distances; the C18…C22, C20…C22, C18A…C22A and C20A…C22A distances; the C…C distances of the methyl carbon atoms of the *tert*-butyl group containing atoms C22 to C25 and C22A to C25A; the C–C distances within the phenyl group C48 to C53 and C48A to C53A. The rigid-bond restraint (RIGU) was applied to the anisotropic

displacement parameters of carbon atoms C12 to C53A (i.e. the entirety of the disordered molecule; 642 restraints). Finally, the SIMU restraint was applied to atoms C12 to C15 and C13A to C15A to improve the quality of the ADPs for the atoms of the two disordered parts (72 restraints). CCDC: 2369785.

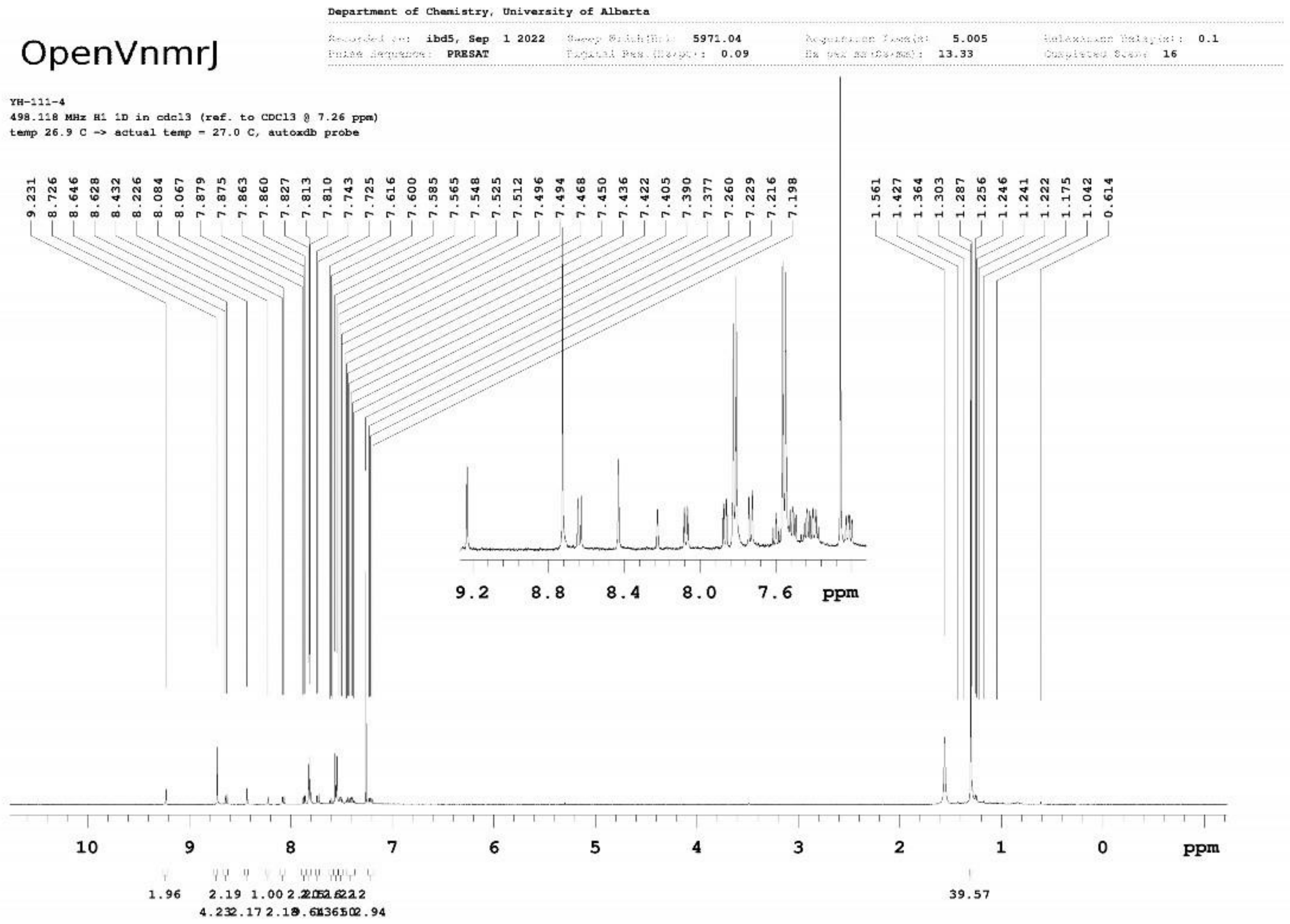

Supplementary Figure 1. ^{1}H NMR spectrum (498 MHz) of mPh(Tc-Py)$_2$ in $CDCl_3$.

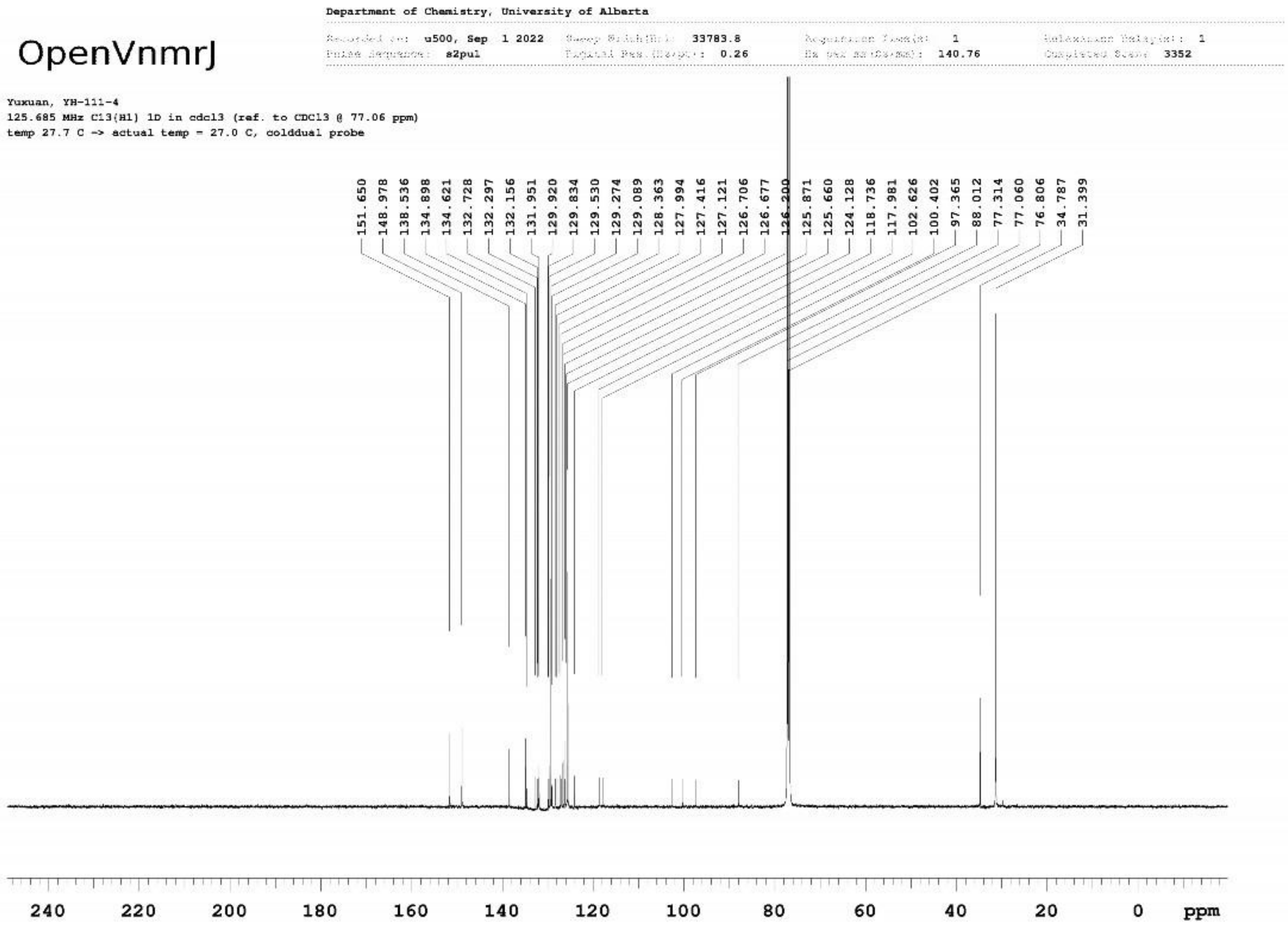

Supplementary Figure 2. ^{13}C NMR spectrum (126 MHz) of mPh(Tc-Py)$_2$ in $CDCl_3$.

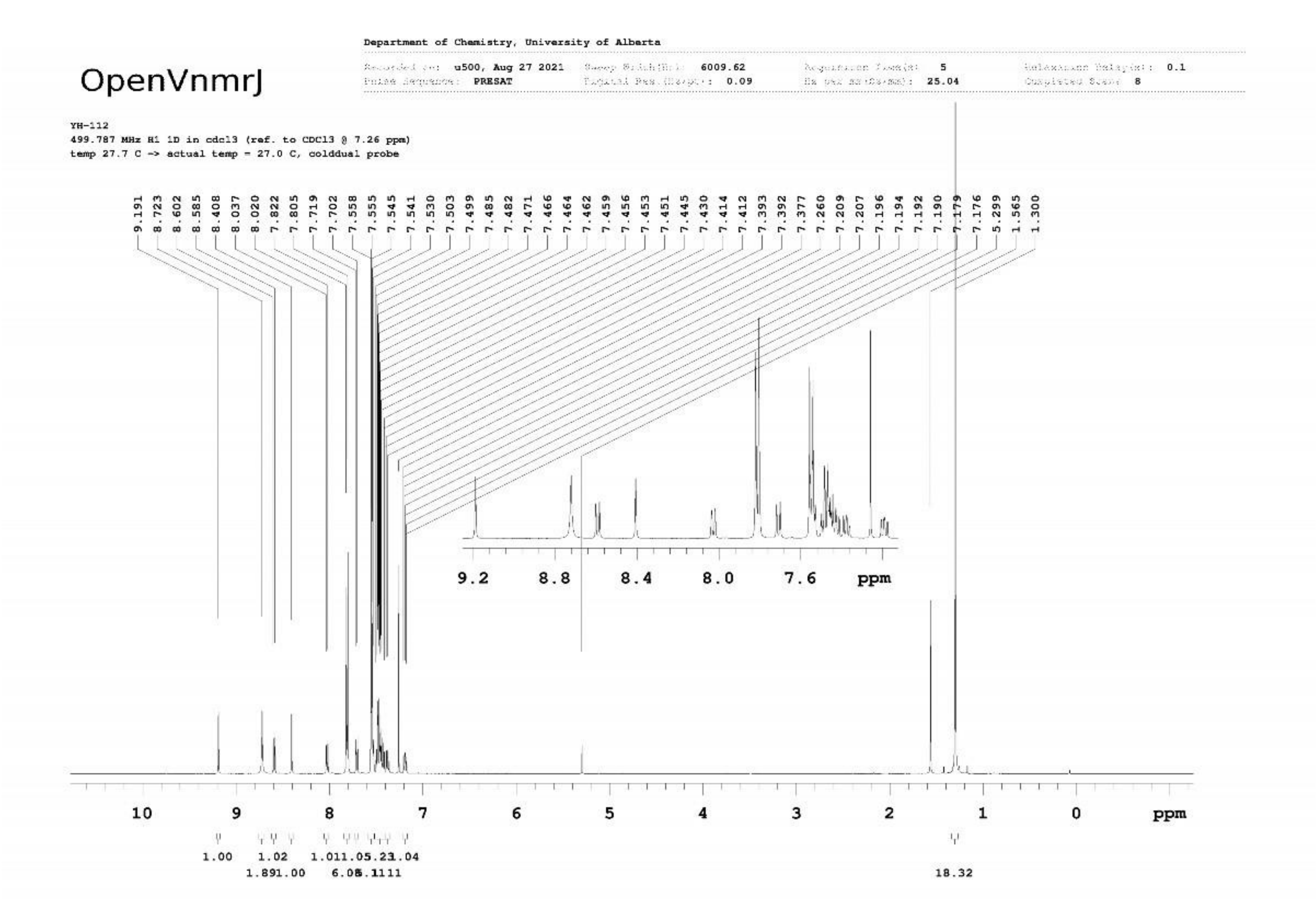

Supplementary Figure 3. ^{1}H NMR spectrum (500 MHz) of Ph(Tc-Py) in $CDCl_3$.

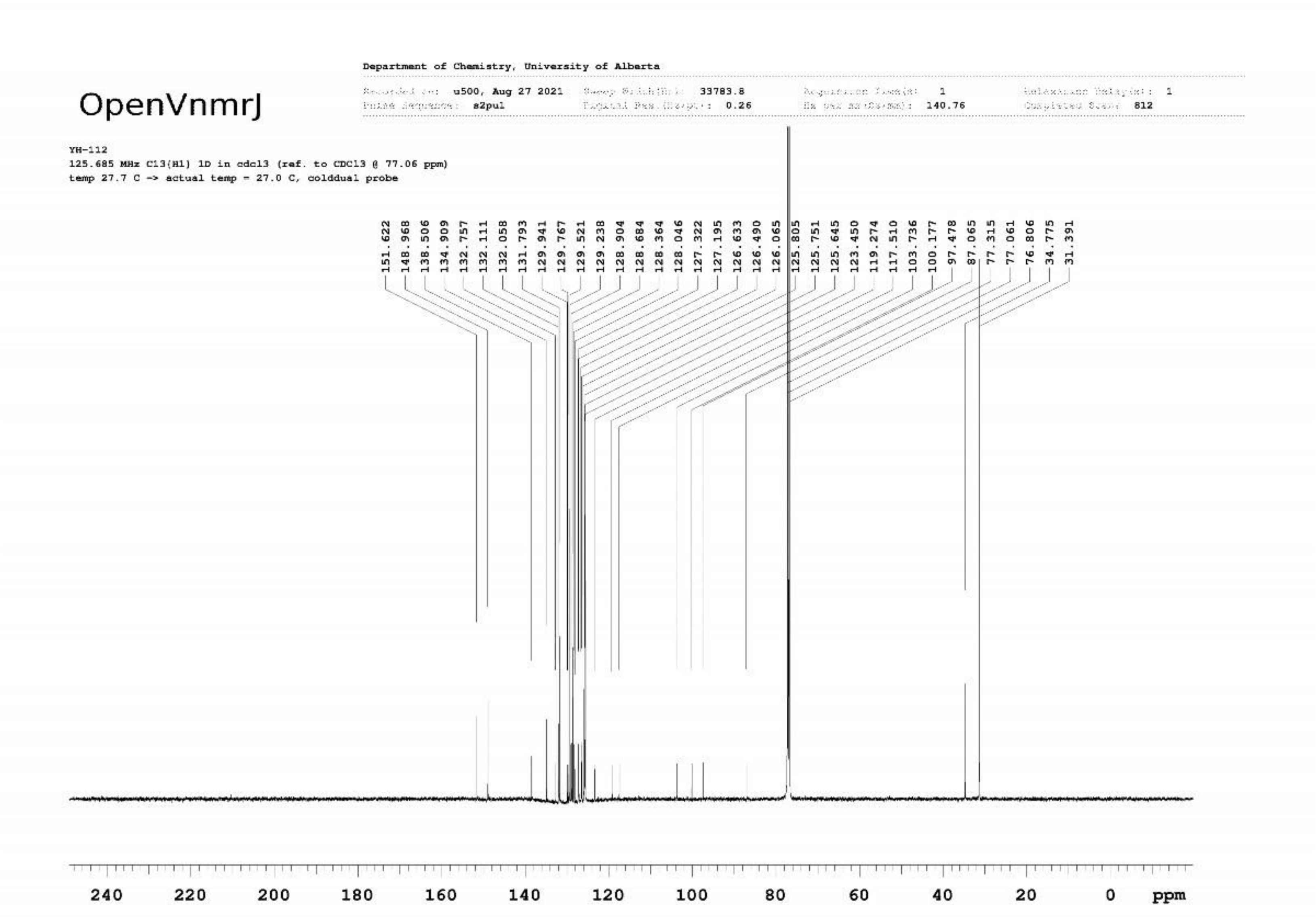

Supplementary Figure 4. ^{13}C NMR spectrum (126 MHz) of Ph(Tc-Py) in $CDCl_3$.

Supplementary Note 2. Photophysical Characterization

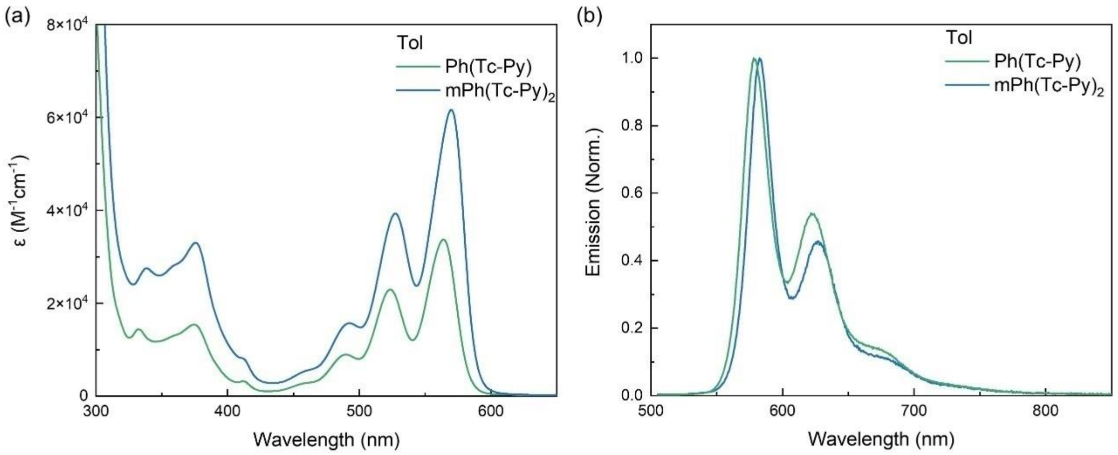

Supplementary Figure 5. Steady-state (a) absorption and (b) fluorescence spectra of Ph(Tc-Py) (green) and mPh(Tc-Py)$_2$ (blue) in toluene.

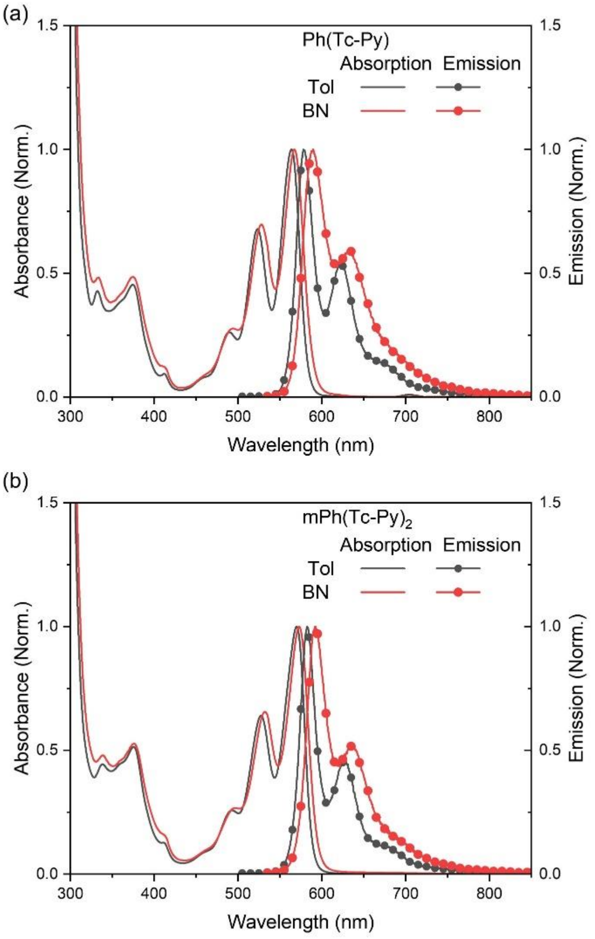

Supplementary Figure 6. Steady-state absorption and fluorescence spectra of (a) Ph(Tc-Py) and (b) mPh(Tc-Py)$_2$ in toluene and benzonitrile.

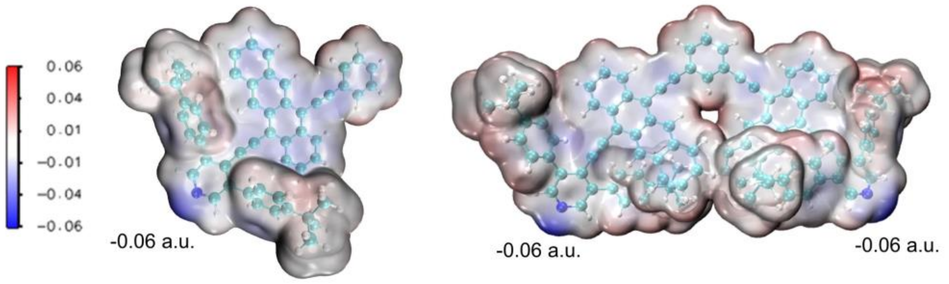

Supplementary Figure 7. The optimized molecular structures and molecular electrostatic potential (MEP) maps of Ph(Tc-Py) and mPh(Tc-Py)$_2$ based on the density functional theory (DFT) method at the B3LYP-GD3BJ/def2svp level of computation.

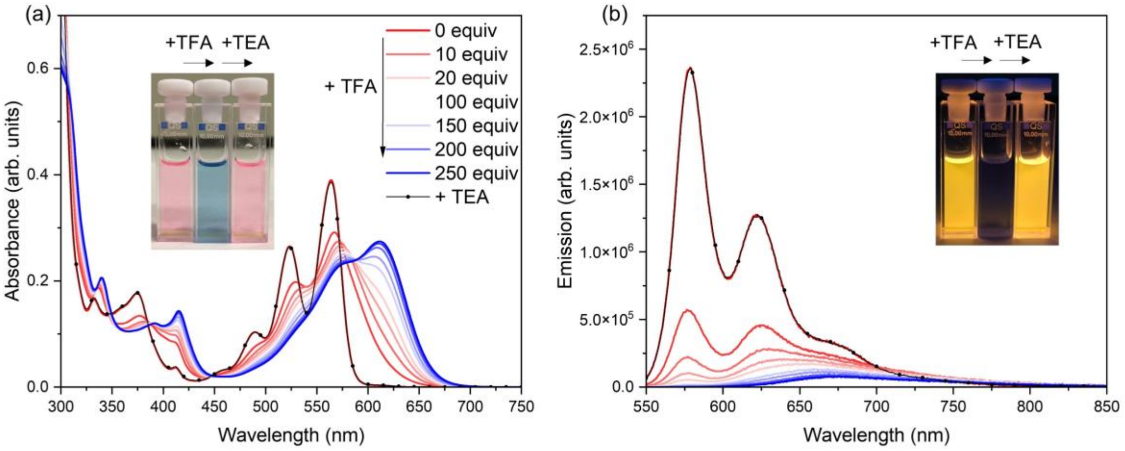

Supplementary Figure 8. Changes to (a) the absorption and (b) fluorescence spectra of Ph(Tc-Py) in toluene (1×10^{-5}M) upon the addition of TFA (from 0 to ca. 250 equivalents).

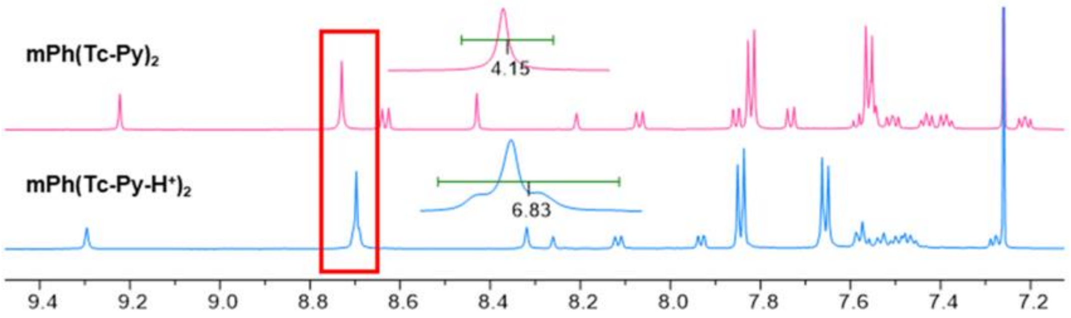

Supplementary Figure 9. Comparison of aromatic region of ^{1}H NMR spectra of mPh(Tc-Py)$_2$ and mPh(Tc-Py-H$^+$)$_2$ (Inset: expansion of peaks in red square with integration).

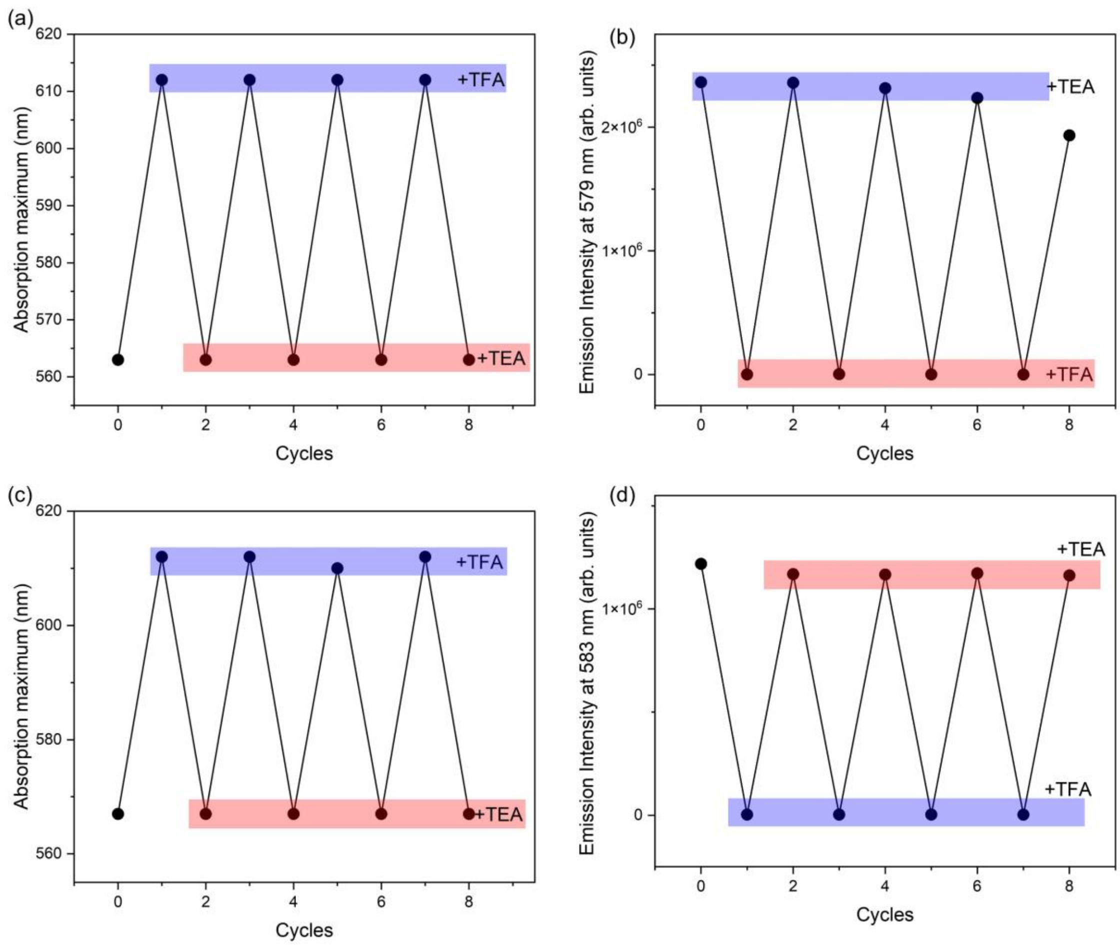

Supplementary Figure 10. Repeated switching of the (a) absorption and (b) fluorescence of Ph(Tc-Py) upon alternate additions of TFA and TEA. Repeated switching of the (c) absorption and (d) fluorescence of mPh(Tc-Py)$_2$ upon alternate additions of TFA and TEA.

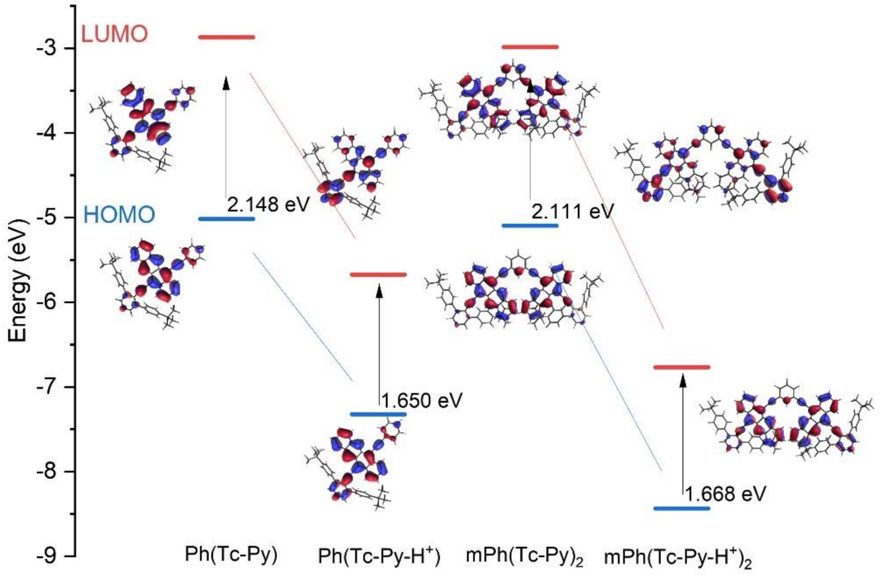

Supplementary Figure 11. Graphic representation of frontier molecular orbitals and respective energies of monomer Ph(Tc-Py) and mPh(Tc-Py)$_2$ and their protonated congeners Ph(Tc-Py-H$^+$) and mPh(Tc-Py-H$^+$)$_2$ as determined by DFT at the B3LYP-GD3BJ/def2svp level.

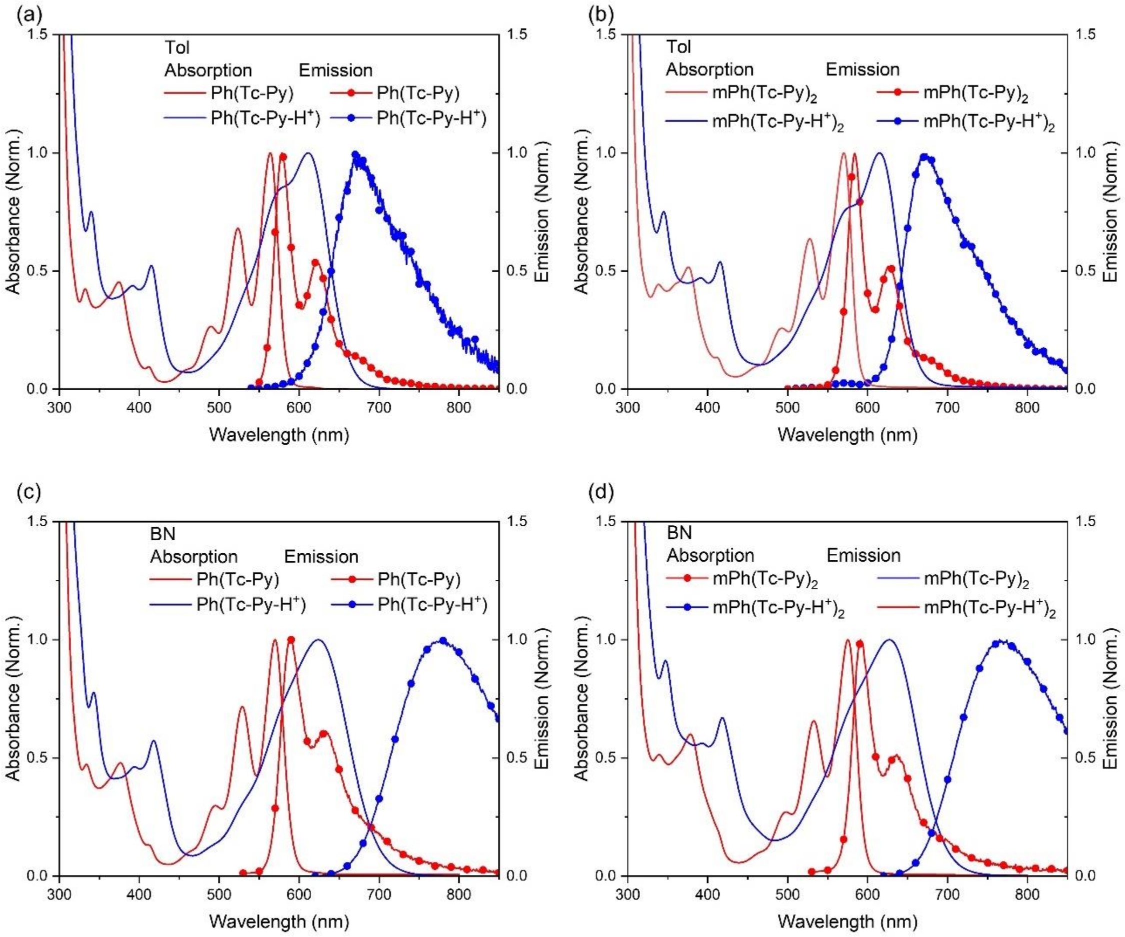

Supplementary Figure 12. Steady-state absorption (solid line) and fluorescence (dotted line) of Ph(Tc-Py) (red) and Ph(Tc-Py-H$^+$) (blue) in (a) toluene and (c) benzonitrile. Steady-state absorption (solid line) and fluorescence (dotted line) of mPh(Tc-Py)$_2$ (red) and mPh(Tc-Py-H$^+$)$_2$ (blue) in (b) toluene and (d) benzonitrile.

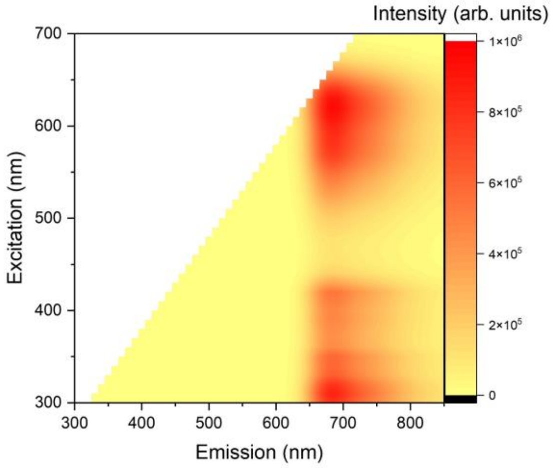

Supplementary Figure 13. Excitation and emission map for mPh(Tc-Py-H^+)$_2$ in toluene.

Supplementary Table 1. Steady-state absorption maxima (λ_{Abs}), fluorescence maxima (λ_{Emi}), Stokes shift (Δ_λ), and fluorescence quantum yields (Φ_F) of Ph(Tc-Py), mPh(Tc-Py)$_2$ and corresponding protonated Ph(Tc-Py)-H^+ and mPh(Tc-Py-H^+)$_2$ forms in toluene and benzonitrile.

Sample	Solvent	λ_{Abs} (nm)	λ_{Emi} (nm)	Δ_λ (nm)	Φ_F[a]
Ph(Tc-Py)	Tol	523, 564	578, 621	14	80%
	BN	529, 570	589, 633	19	89%
mPh(Tc-Py)$_2$	Tol	528, 570	583, 627	13	28%
	BN	532, 575	591, 636	16	51%
Ph(Tc-Py-H^+)	Tol	611	673	62	20%
	BN	623	776	153	
mPh(Tc-Py-H^+)$_2$	Tol	615	671	56	16%
	BN	629	768	139	

[a]Samples were measured under ambient conditions by an absolute method using an integrating sphere. An error margin of ±20% is implicit in the determination of Φ_F.

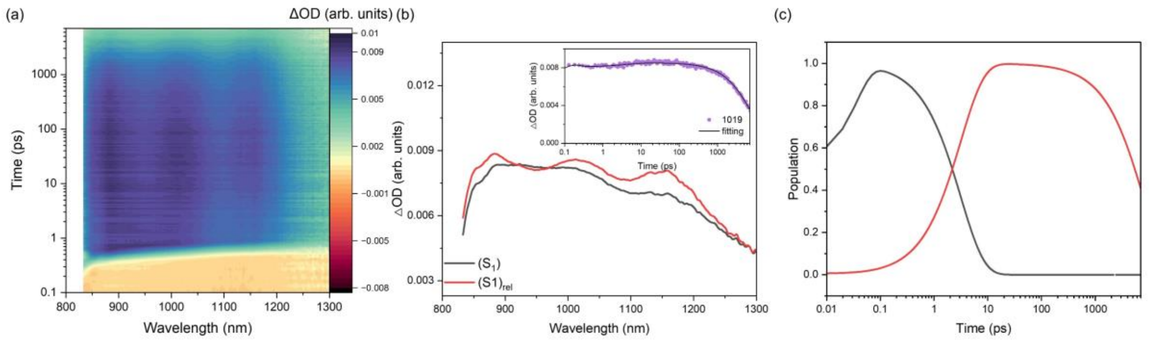

Supplementary Figure 14. Femtosecond transient absorption spectroscopy in near-infrared region of Ph(Tc-Py) in argon-saturated toluene at room temperature. (a) Heat map of the recorded differential absorption spectra at various time delays between 0 to 7000 ps after photoexcitation at 510 nm. (b) Evolution-associated spectra (EAS) and insert that depicts time absorption profile as well as corresponding fit of selected wavelength (see Figure legend for details). (c) The relative population of the respective species with colors correlating with the evolution-associated spectra (EAS).

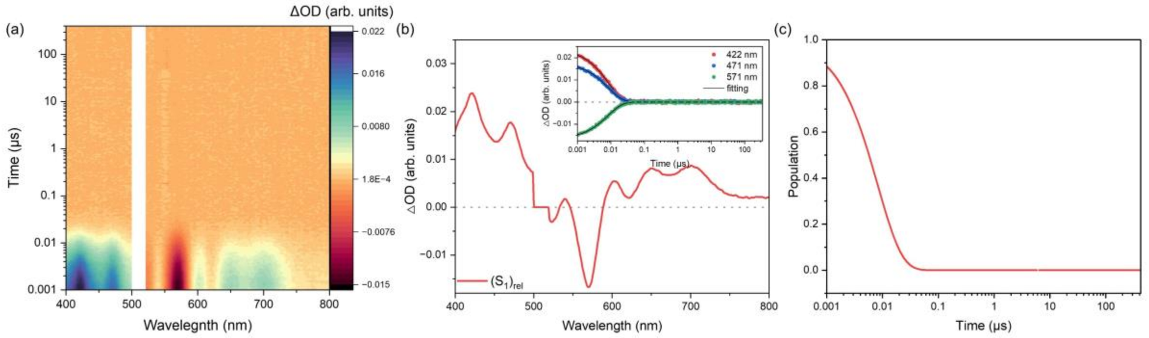

Supplementary Figure 15. Nanosecond transient absorption spectroscopy in visible region of Ph(Tc-Py) in argon-saturated toluene at room temperature. (a) Heat map of the recorded differential absorption spectra at various time delays between 0 to 330 μs after photoexcitation at 510 nm. (b) Evolution-associated spectra (EAS) and insert that depicts time absorption profiles as well as corresponding fits of selected wavelengths (see Figure legend for details). (c) The relative population of the respective species with colors correlating with the evolution-associated spectra (EAS).

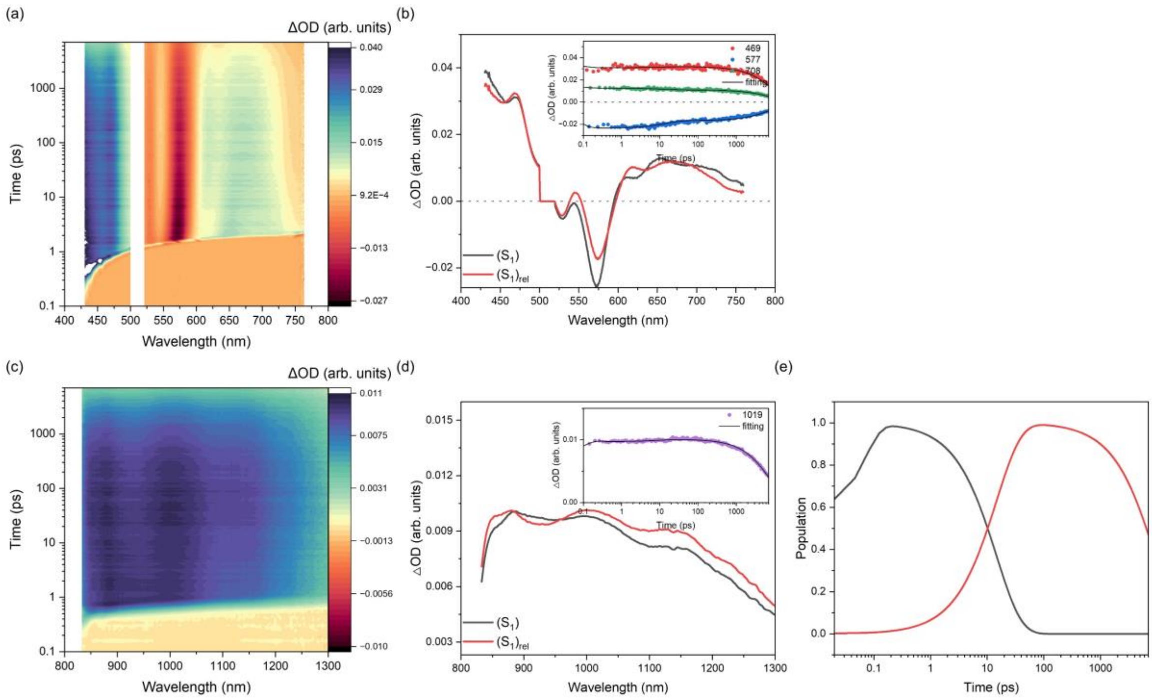

Supplementary Figure 16. Femtosecond transient absorption spectroscopy of Ph(Tc-Py) in argon-saturated benzonitrile at room temperature in the (a,b) visible and (c,d) near-infrared region. (a,c) Heat map of the recorded differential absorption spectra at various time delays between 0 to 7000 ps after photoexcitation at 510 nm. (b,d) Evolution-associated spectra (EAS) and insert that depicts time absorption profiles as well as corresponding fits of selected wavelengths (see Figure legend for details). (e) The relative population of the respective species with colors correlating with the evolution-associated spectra (EAS).

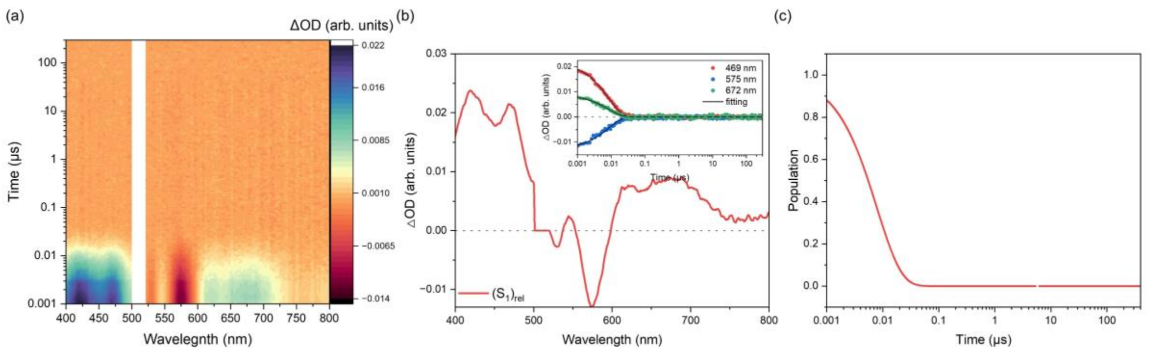

Supplementary Figure 17. Nanosecond transient absorption spectroscopy in visible region of Ph(Tc-Py) in argon-saturated benzonitrile at room temperature. (a) Heat map of the recorded differential absorption spectra at various time delays between 0 to 330 μs after photoexcitation at 510 nm. (b) Evolution-associated spectra (EAS) and insert that depicts time absorption profiles as well as corresponding fits of selected wavelengths (see the figure legend for details). (c) The relative population of the respective species with colors correlating with the evolution-associated spectra (EAS).

Supplementary Table 2. Summary of photoluminescence behavior of Ph(Tc-Py) in toluene and benzonitrile.

Ph(Tc-Py)	Φ_F	$\tau\,(S_1)_{rel}$ (ns)	k_r (s^{-1})	k_{nr} (s^{-1})
toluene	0.80 (±10%)	8.2 (±10%)	9.7×10^7 (±14%)	2.4×10^7 (±14%)
benzonitrile	0.89 (±10%)	7.9 (±10%)	11.0×10^7 (±14%)	1.4×10^7 (±14%)

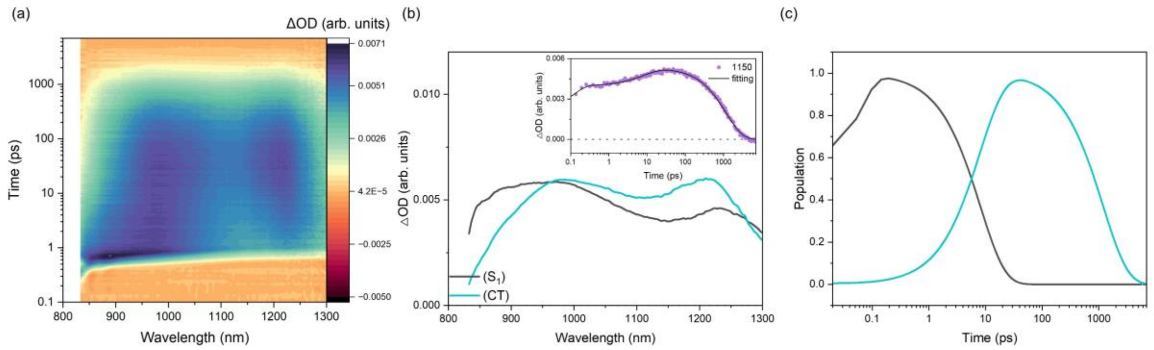

Supplementary Figure 18. Femtosecond transient absorption spectroscopy in near-infrared region of Ph(Tc-Py-H$^+$) in argon-saturated toluene at room temperature. (a) Heat map of the recorded differential absorption spectra at various time delays between 0 to 7000 ps after photoexcitation at 510 nm. (b) Evolution-associated spectra (EAS) and insert that depicts time absorption profile as well as corresponding fit of selected wavelength (see Figure legend for details). (c) The relative population of the respective species with colors correlating with the evolution-associated spectra (EAS).

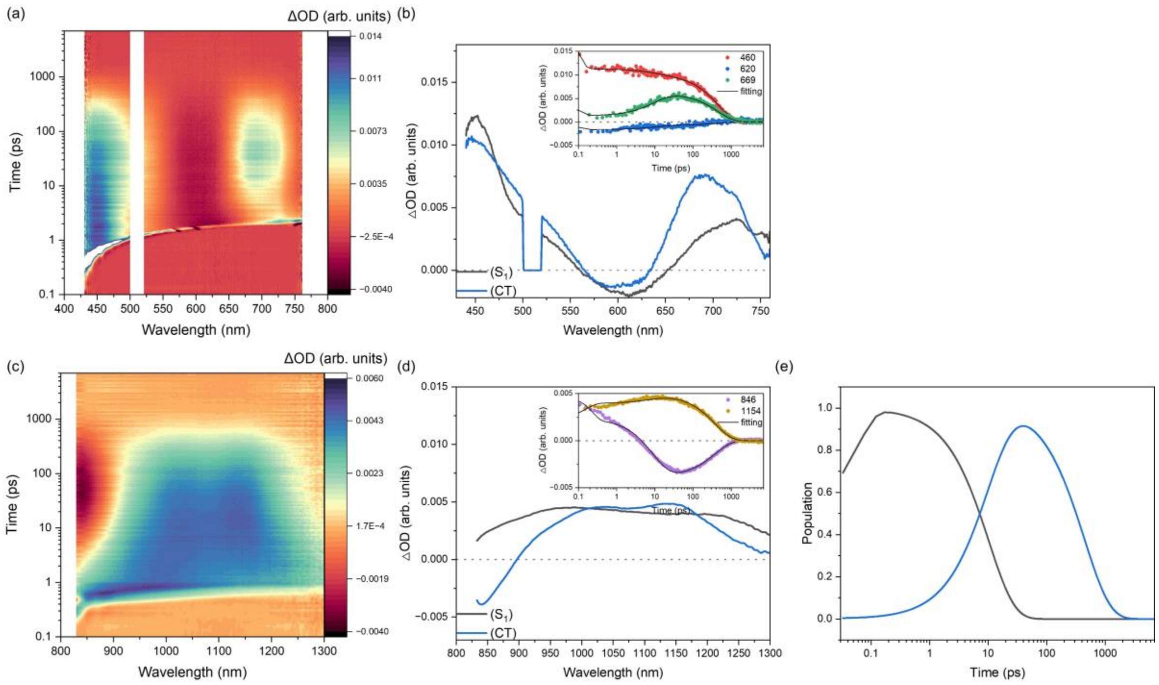

Supplementary Figure 19. Femtosecond transient absorption spectroscopy of Ph(Tc-Py-H^{+}) in argon-saturated benzonitrile at room temperature in the (a,b) visible and (c,d) near-infrared region. (a,c) Heat map of the recorded differential absorption spectra at various delays between 0 to 7000 ps after photoexcitation at 510 nm. (b,d) Evolution-associated spectra (EAS) and insert that depicts time absorption profiles as well as corresponding fits of selected wavelengths (see Figure legend for details). (e) The relative population of the respective species with colors correlating with the evolution-associated spectra (EAS).

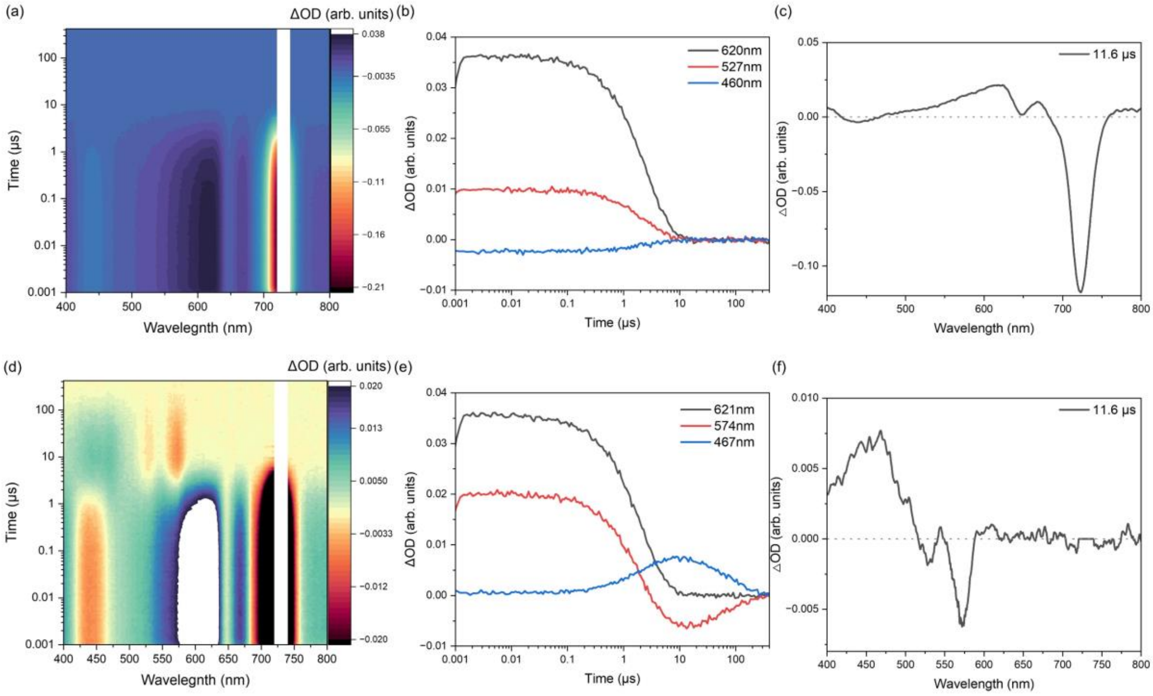

Supplementary Figure 20. (a-c) Intersystem crossing of $PdPc(OBu)_8$ in argon-saturated toluene. (d-e) Triplet-triplet sensitization of $mPh(Tc\text{-}Py)_2$ in argon-saturated toluene. (a) Heat map of the recorded differential absorption spectra at various time delays between 0 to 400 μs after photoexcitation at 730 nm. (b) The time absorption profile illustrates the dynamics of the triplet excited state of $PdPc(OBu)_8$. (c) The differential absorption spectrum of the data at a time delay of 1.4 μs represents the characteristic signature of the triplet excited state of $PdPc(OBu)_8$. (d) Heat map of the recorded differential absorption spectra at various time delays between 0 to 400 μs after photoexcitation at 730 nm. (e) Time absorption profiles of the spectra illustrate the dynamics of the triplet excited state of $PdPc(OBu)_8$ transduction of the triplet excited-state energy to $mPh(Tc\text{-}Py)_2$. (f) The differential absorption spectrum of data at a time delay of 11.6 μs represents the triplet excited-state absorption spectrum of $mPh(Tc\text{-}Py)_2$.

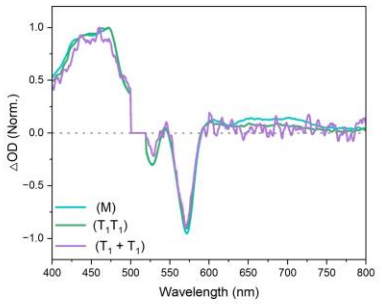

Supplementary Figure 21. Normalized evolution-associated spectra (EAS) of the respective species for nanosecond transient absorption spectroscopy of mPh(Tc-Py)$_2$ in argon-saturated toluene at room temperature.

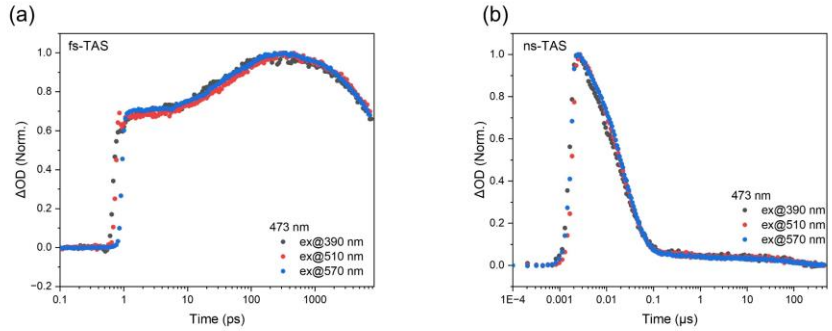

Supplementary Figure 22. Triplet evolution dynamics of mPh(Tc-Py)$_2$ in argon-saturated toluene at room temperature at photoexcitation wavelengths of 390, 510, and 570 nm. (a) single-wavelength kinetics at 473 nm in femtosecond transient absorption spectroscopy. (b) single-wavelength kinetics at 473 nm in nanosecond transient absorption spectroscopy.

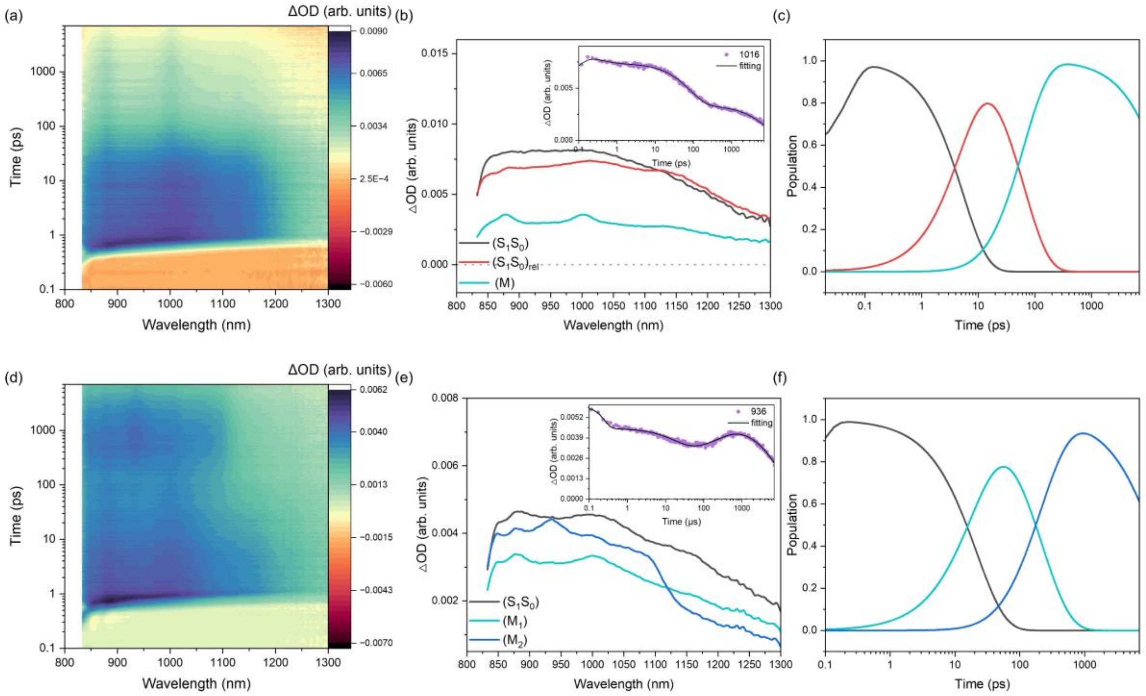

Supplementary Figure 23. Femtosecond transient absorption spectroscopy in near-infrared region of mPh(Tc-Py)2 in (a–c) argon-saturated toluene at room temperature and (d–f) argon-saturated benzonitrile at room temperature. (a and d) Heat map of the recorded differential absorption spectra at various time delays between 0 to 7000 ps after photoexcitation at 510 nm. (b and e) Evolution-associated spectra (EAS) and insert that depicts time absorption profile as well as corresponding fit of selected wavelength (see the figure legend for details). (c and f) The relative population of the respective species with colors correlating with the evolution-associated spectra (EAS).

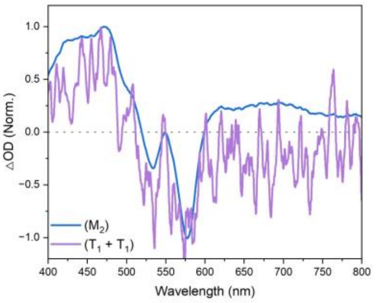

Supplementary Figure 24. Normalized evolution-associated spectra (EAS) of the respective species for nanosecond transient absorption spectroscopy of mPh(Tc-Py)2 in argon-saturated benzonitrile at room temperature.

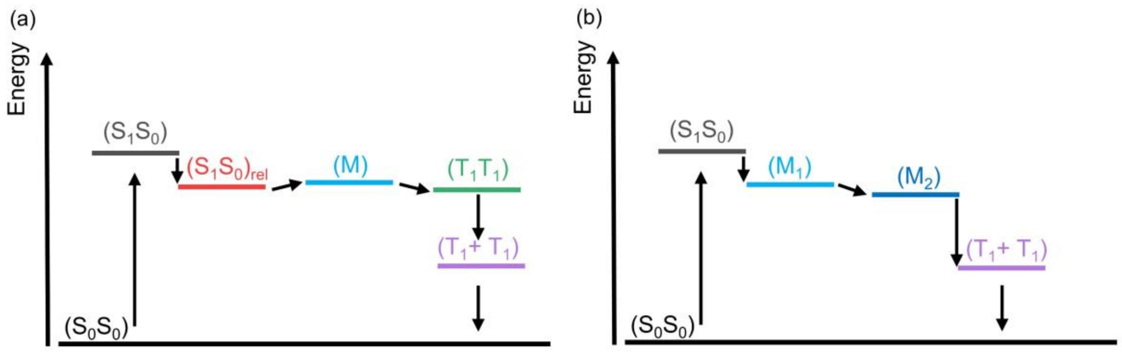

Supplementary Figure 25. Qualitative energy diagram showing the sequential deactivation cascade for mPh(Tc-Py)$_2$ in (a) toluene and (b) benzonitrile after excitation at 510 nm. For mPh(Tc-Py)$_2$ in toluene, initial photoexcitation into (S_1S_0) is followed by solvent relaxation to produce $(S_1S_0)_{rel}$, which is subsequently converted into a mixed state (M) which is a combination of the $(S_1S_0)_{rel}$, CT, and (T_1T_1) states. Then, the correlated triplet pair (T_1T_1) is formed via the mixed state. Next, the spin decoherence of (T_1T_1) yields free triplet excited states (T_1+T_1). For mPh(Tc-Py)$_2$ in benzonitrile, the initial excitation into (S_1S_0) is followed by the formation of an initial mixed state (M_1). Subsequently, another mixed state (M_2), with the more intense signature of the CT state, is formed. Next, a free triplet excited state (T_1+T_1) is followed without intermediation of a pure (T_1T_1) state.

Supplementary Table 3. Summary of lifetimes obtained from Global Analysis of femtosecond and nanosecond transient absorption (fs- and ns-TAS) measurements of Ph(Tc-Py), mPh(Tc-Py)$_2$, and corresponding protonated forms Ph(Tc-Py)-H$^+$ and mPh(Tc-Py-H$^+$)$_2$ in toluene and benzonitrile. Singlet oxygen quantum yield (Φ_Δ) of mPh(Tc-Py)$_2$ in toluene and benzonitrile. For parameters determined via transient absorption measurements, an error in the range of ±10% is considered.

Sample	Solvent	fs-TAS		ns-TAS			Φ_Δ
		τ	τ	τ	τ	τ	
Ph(Tc-Py)	Tol	(S_1) 3.1 ps	-	$(S_1)_{rel}$ 8.2 ns	-	-	-
	BN	(S_1) 14.6 ps	-	$(S_1)_{rel}$ 7.9 ns	-	-	-
mPh(Tc-Py)$_2$	Tol	(S_1S_0) 5.4 ps	$(S_1S_0)_{rel}$ 64.5 ps	(M) 8.3 ns	(T_1T_1) 29.7 ns	($T_1 + T_1$) 77.5 µs	113%
	BN	(S_1S_0) 21.7 ps	(M_1) 220.0 ps		(M_2) 13.0 ns	($T_1 + T_1$) 94.0 µs	82%
Ph(Tc-Py-H$^+$)	Tol	(S_1) 8.2 ps	CT 1.2 ns	-	-	-	-
	BN	(S_1) 10.3 ps	CT 445.7 ps	-	-	-	-
mPh(Tc-Py-H$^+$)$_2$	Tol	(S_1) 8.4 ps	CT 1.7 ns	-	-	-	-
	BN	(S_1) 10.2 ps	CT 390 ps	-	-	-	-

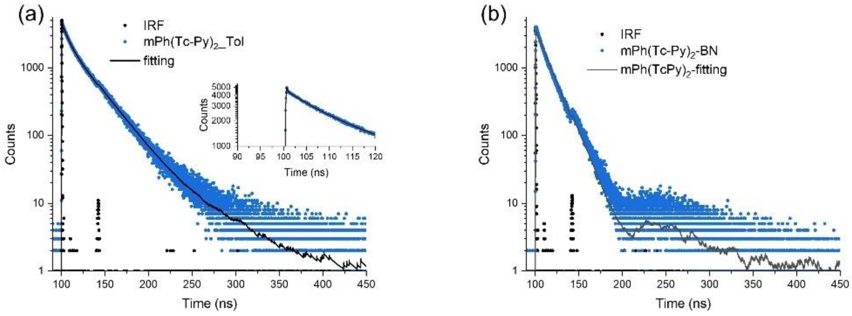

Supplementary Figure 26. TCSPC data of mPh(Tc-Py)$_2$ in argon-purged (a) toluene and (b) benzonitrile and fitting thereof.

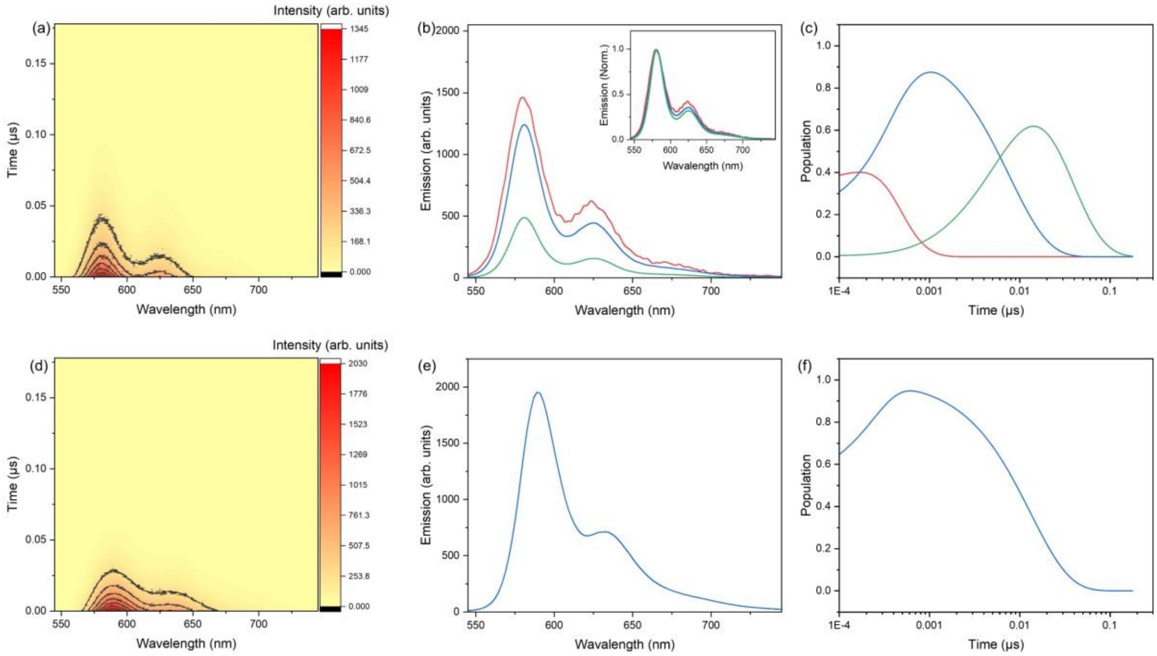

Supplementary Figure 27. TRES raw data and corresponding global analysis for mPh(Tc-Py)$_2$, following photoexcitation at 510 nm in argon-saturated (a–c) toluene and (d–f) benzonitrile at room temperature. (a and d) Heat map of the recorded TRES at various delays between 0 to 200 ns after photoexcitation at 510 nm. (b and e) Evolution-associated spectra (EAS); The normalized EAS in toluene are exhibited in the inset. (c and f) Relative populations of the respective species with colors correlating with the evolution-associated spectra (EAS)

Supplementary Table 4. Lifetimes (τ) as well as relative amplitudes of TCSPC and the fitting results of time resolve emission spectra (TRES) of mPh(Tc-Py)$_2$ in argon-purged toluene and benzonitrile.

		TCSPC			TRES		
	Solvent	τ_1	τ_2	τ_3	τ_1	τ_2	τ_3
mPh(Tc-Py)$_2$	Toluene	<200 ps (0.98%)	7.4 ns (18.86%)	27.4 ns (80.16%)	<200 ps	7.7 ns	29.0 ns
	Benzonitrile			13.6 ns (100%)			13.2 ns

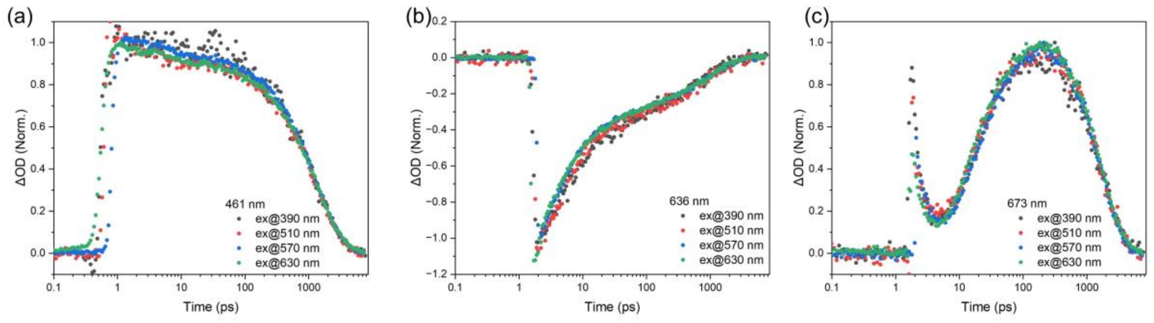

Supplementary Figure 28. Femtosecond transient absorption spectroscopy of mPh(Tc-Py-H$^+$)$_2$ in argon-saturated toluene at different photoexcitation wavelengths of 390, 510, 570, and 630 nm: single wavelength kinetics at (a) 461, (b) 636, and (c) 673 nm.

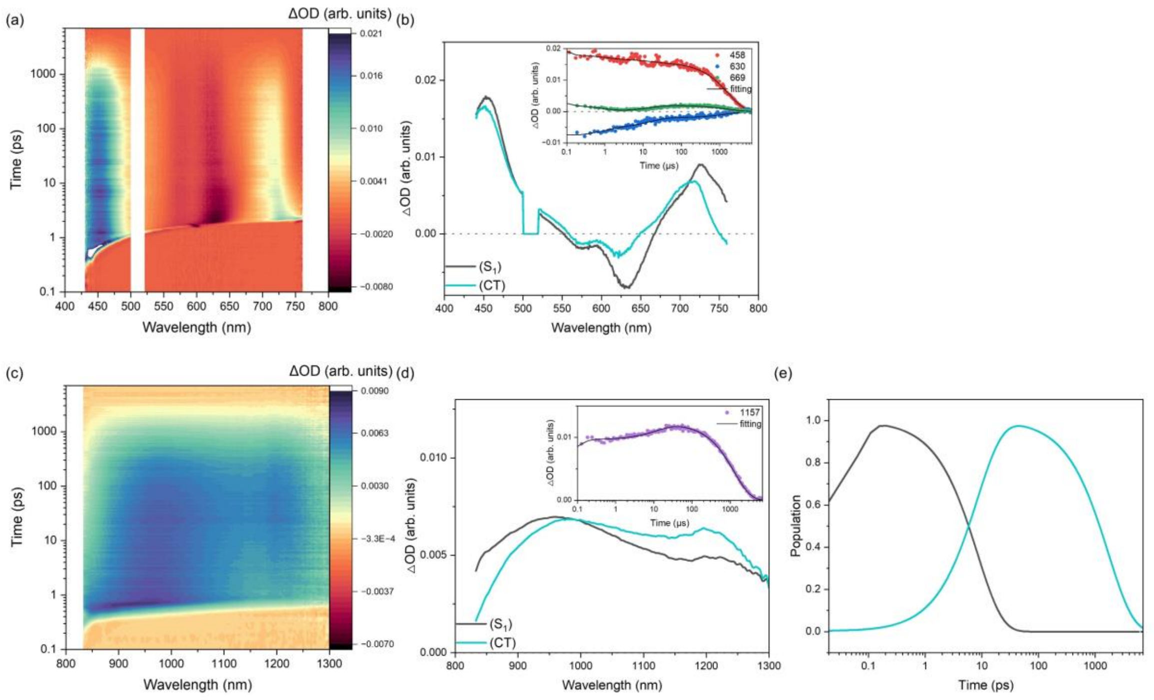

Supplementary Figure 29. Femtosecond transient absorption spectroscopy of mPh(Tc-Py-H$^+$)$_2$ in argon-saturated toluene at room temperature. (a and c) Heat map of the recorded differential absorption spectra at various time delays between 0 to 7000 ps after photoexcitation at 510 nm. (b and d) Evolution-associated spectra (EAS) and insert that depicts time absorption profiles as well as corresponding fits of selected wavelengths (see Figure legend for details). (e) The relative population of the respective species with colors correlating with the evolution-associated spectra (EAS).

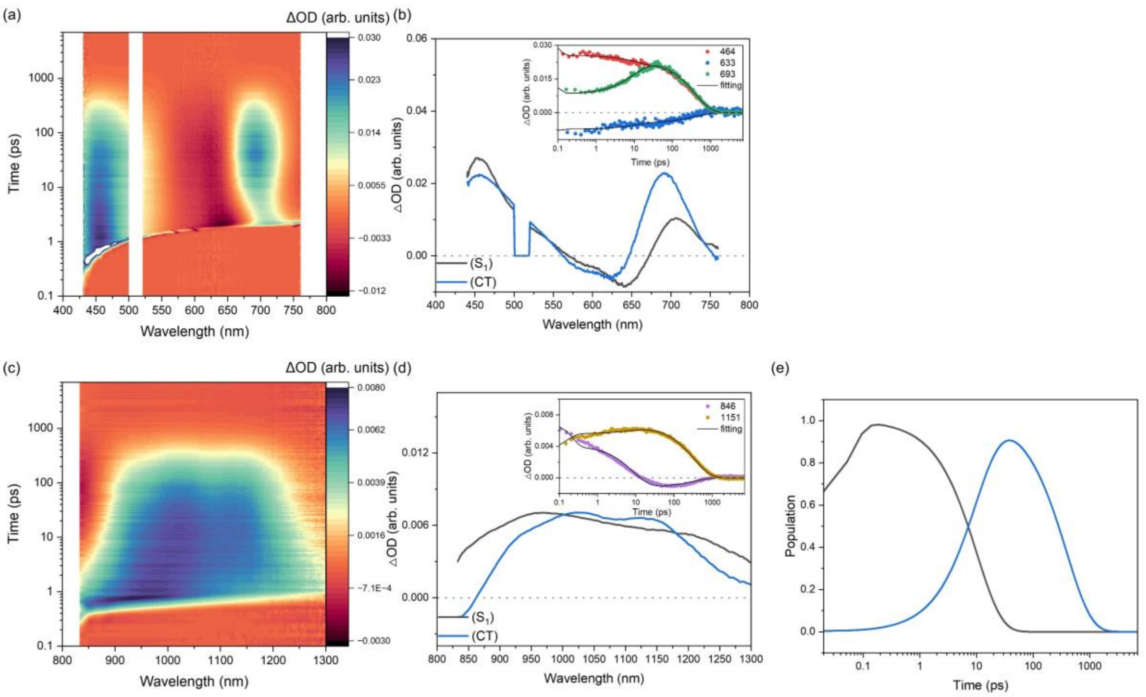

Supplementary Figure 30. Femtosecond transient absorption spectroscopy of mPh(Tc-Py-H^{+})$_2$ in argon-saturated benzonitrile at room temperature. (a and c) Heat map of the recorded differential absorption spectra at various time delays between 0 to 7000 ps after photoexcitation at 510 nm. (b and d) Evolution-associated spectra (EAS) and insert that depicts time absorption profiles as well as corresponding fits of selected wavelengths (see Figure legend for details). (e) The relative population of the respective species with colors correlating with the evolution-associated spectra (EAS).

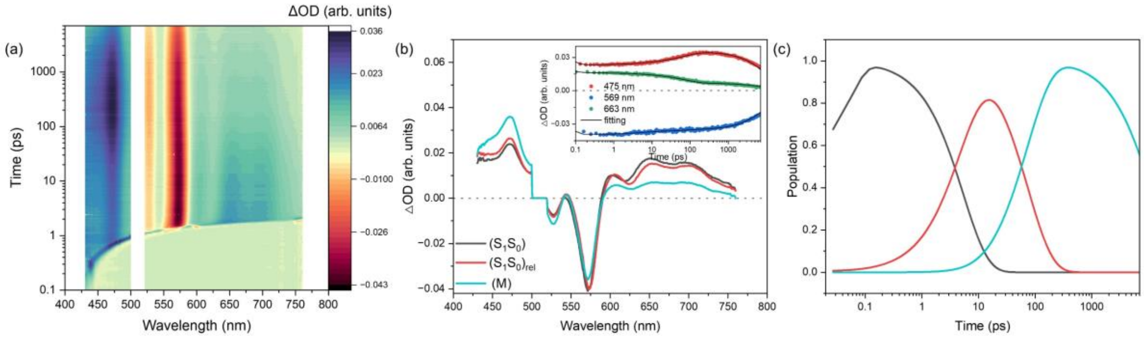

Supplementary Figure 31. Femtosecond transient absorption spectroscopy of mPh(Tc-Py)$_2$ in argon-saturated toluene at room temperature after sequentially adding TFA and TEA. (a) Heat map of the recorded differential absorption spectra at various time delays between 0 to 7000 ps after photoexcitation at 510 nm. (b) Evolution-associated spectra (EAS) and insert that depicts time absorption profiles as well as corresponding fits of selected wavelengths (see Figure legend for details). (c) The relative population of the respective species with colors correlating with the evolution-associated spectra (EAS).

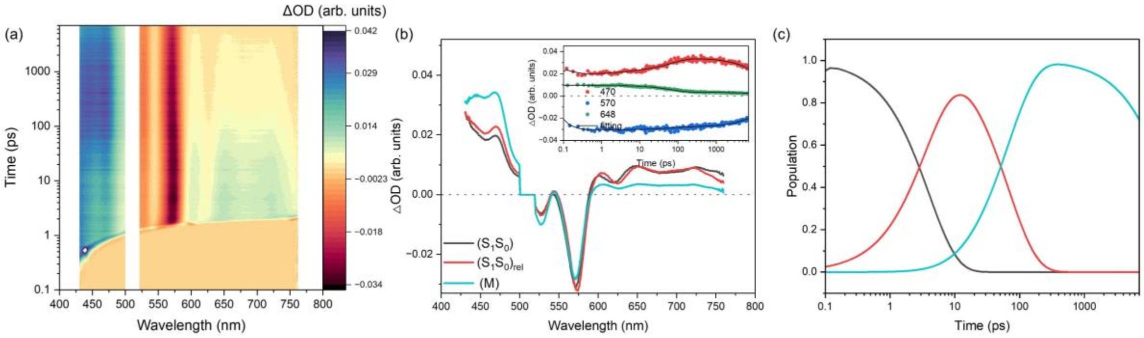

Supplementary Figure 32. Femtosecond transient absorption spectroscopy of mPh(Tc-Py)$_2$ in argon-saturated toluene at room temperature after adding TEA. (a) Heat map of the recorded differential absorption spectra at various time delays between 0 to 7000 ps after photoexcitation at 510 nm. (b) Evolution-associated spectra (EAS) and insert that depicts time absorption profiles as well as corresponding fits of selected wavelengths (see Figure legend for details). (c) The relative population of the respective species with colors correlating with the evolution-associated spectra (EAS).

Supplementary Note 3. Supplementary References

1. Eidamshaus, C. & Reissig, H. U. A chiral pool strategy for the synthesis of enantiopure hydroxymethyl-substituted pyridine derivatives. *European J. Org. Chem.* 6056–6069 (2011) doi:10.1002/ejoc.201100681.
2. Gao, Y. *et al.* The loss of endgroup effects in long pyridyl-endcapped oligoynes on the way to carbyne. *Nat. Chem.* **12**, 1143–1149 (2020).
